# Periodontal Health Status and Associated Factors: Findings of a Prenatal Oral Health Program in South Brazil

**DOI:** 10.1155/2017/3534048

**Published:** 2017-03-29

**Authors:** Marta Silveira da Mota Krüger, Renata Picanço Casarin, Letycia Barros Gonçalves, Fernanda Geraldo Pappen, Fernanda Oliveira Bello-Correa, Ana Regina Romano

**Affiliations:** Graduate Program in Dentistry, Federal University of Pelotas (UFPel), Pelotas, RS, Brazil

## Abstract

*Objective.* The aims of this study were to evaluate the periodontal health of pregnant women and to investigate the association of periodontal status with demographic and socioeconomic characteristics, as well as medical and dental history.* Materials and Methods.* A total of 311 pregnant women were interviewed to obtain sociodemographic data along with medical and dental histories. Clinical examinations were performed to record the presence of visible plaque, gingival bleeding, and caries activity. The periodontal condition was evaluated by Community Periodontal Index of Treatment Needs (CPITN) in one tooth of each sextant (16, 11, 26, 36, 31, and 46).* Results.* After the adjustment analysis, the presence of visible plaque remained the main determinant of gingival bleeding (OR = 2.91, CI = 1.91–4.48). First-trimester pregnancy status was also a predictor, with a lower prevalence of gingival bleeding observed in the second (OR = 0.87, CI = 0.77–0.99) and third (OR = 0.82, CI = 0.73–0.93) trimesters.* Conclusion.* In pregnant women, the presence of dental plaque and first-trimester pregnancy status were the main implicated factors predicting gingival bleeding.

## 1. Introduction

Periodontal diseases are chronic infections related to Gram-negative bacteria [1]. The most recent classification of periodontal diseases from the American Association of Periodontology in 1999 included “pregnancy gingivitis” as an entity [2]. Gingivitis is the most prevalent periodontal condition during pregnancy [3], affecting from 36% to 100% of pregnant women [4–6].

Although not thought to cause periodontal disease, pregnancy may exacerbate preexisting periodontal conditions [7]. A systematic review concluded that gingival inflammation is significantly increased during pregnancy, without a concomitant increase in biofilm levels [8]. However, due to decreased hormone production during the postpartum phase, gingivitis disappears [9–12], with no permanent effects on periodontal attachment [5, 9, 10, 12–15]. This association may lead to an overestimation of the effects of pregnancy on gingival inflammation, as hormones are well-known irritants of previously inflamed gingival tissue [16].

The precise effects of pregnancy on healthy gingiva have not been well-established [8]. Increased circulating levels of estrogen and progesterone are believed to affect the development of localized inflammation by stimulating prostaglandin production [13, 17] and affecting the immune response [18]. Alterations in the composition of the subgingival biofilm have been reported in the gestational period [19]. Some authors have proposed that circulating hormones during pregnancy increase the prevalence of* Prevotella intermedia* and* Porphyromonas gingivalis*, bacterial species usually associated with gingival inflammation [20, 21]. The higher prevalence of such species has been positively correlated with salivary hormone levels [22].

The association of sociodemographic characteristics with periodontal conditions during pregnancy has also been investigated. Low socioeconomic status has been associated with diminished access to health services and unawareness of oral hygiene habits [23]. Some studies have also reported a significant association between the occurrence of pregnancy gingivitis and unemployment and low educational levels [3, 23, 24]. Therefore, the aims of this study were to evaluate the periodontal status of pregnant women and to investigate the association of periodontal status with demographic and socioeconomic characteristics, as well as medical and dental history.

## 2. Study Population and Methodology

The study population of this observational study consisted of women in all stages of pregnancy, assisted in the Federal University of Pelotas Prenatal Oral Health Program, a spinoff of a program promoting oral health in infants. A total of 311 patients were included in the study.

All participants provided written informed consent prior to study participation. The study was approved by the institutional ethics committee (protocol number 214/2011). Pregnant women were interviewed to obtain sociodemographic data, such as age, educational level, employment status, family income, and marital and parity status. Medical and dental histories were also collected, including oral hygiene habits, such as tooth brushing and flossing frequency.

The clinical examinations were performed by previously calibrated examiners at the dental care clinic of the Federal University of Pelotas Prenatal Oral Health Program. The presence of visible plaque [25], gingival bleeding (at least one site of bleeding after the use of dental floss by the examiner), and caries activity were recorded; the characteristics of carious lesions (active/inactive) were classified following Nyvad et al. [26]. The periodontal condition was also assessed using the Community Periodontal Index of Treatment Needs (CPITN) index [27]. The CPITN assessment was performed in one tooth of each sextant (16, 11, 26, 36, 31, and 46), using a millimeter periodontal probe.

Data were entered twice into an Epi Info 6 (Centers for Disease Control and Prevention, Atlanta, GA, USA) database. Their consistency and range were checked automatically. Statistical analyses were performed using Stata software (version 10.0 for Windows; Stata Corp. LP, College Station, TX, USA). Descriptive statistics were generated by calculating means and standard deviations of continuous variables and frequencies and percentages of categorical variables. The independent variables (age, income, education, stage of pregnancy, prenatal care, frequency of tooth brushing and flossing, presence of visible plaque, and caries activity) were included in a Poisson regression with robust variance analysis. To adjust the analysis, variables that did not contribute to the model were removed and a new model was constructed.

## 3. Results

All recruited pregnant women agreed to participate in the study (*n* = 311; 100% response rate). The sociodemographic and obstetric profiles of the study population are shown in Table 1. Most participants were aged 20–34 years (70.4%), the major occupation (55.2%) of participants was “housewife,” and 41.4% of monthly family incomes were in the range of US $351–1050. Regarding education level, 52.7% of participants reported having more than 8 years of education. A total of 33 women (10.6%) first enrolled in the Prenatal Oral Health Program in their first trimester, while 154 (49.5%) and 124 (39.9%) enrolled in the second and third trimesters, respectively.

Oral hygiene habits, such as tooth brushing and flossing frequency, and the prevalence of visible plaque, gingival bleeding, caries activity, and CPITN score are described in Table 2. Associations of gingival bleeding during pregnancy with other independent variables are shown in Table 3. Poisson regression analysis indicated that gingival bleeding occurrence in pregnant women was associated with family income (US $351–1050: OR = 1.30, CI = 1.11–1.52; <US$ 350: OR = 1.25, CI = 1.06–1.48), skin color (OR = 1.13, CI = 1.04–1.24), prenatal care (OR = 1.19, CI = 1.13–1.25), presence of visible plaque (OR = 2.87, CI = 1.92–4.30), and caries activity (OR = 1.44, CI = 1.23–1.69). Second- (OR = 0.87, CI = 0.79–0.96) and third-trimester (OR = 0.83, CI = 0.75–0.93) pregnancy status were associated with less gingival bleeding compared to first-trimester pregnancy status. After adjustment, presence of visible plaque remained the main determinant of gingival bleeding (OR = 2.79, CI = 1.81–4.30). First-trimester pregnancy status was also a determinant of gingival bleeding, with a lower prevalence observed in the second (OR = 0.89, CI = 0.78–1.01) and third (OR = 0.83, CI = 0.73–0.95) trimesters of pregnancy (Table 3).

Associations of CPITN scores in pregnant women with other independent variables are shown in Table 4. Poisson regression analysis indicated that deeper periodontal pockets in pregnant women were associated with skin color (OR = 1.25, CI = 1.10–1.41), family income (US $351–1050: OR = 1.22, CI = 1.04–1.44; <US$ 350: OR = 1.36, CI = 1.16–1.60), education level (OR = 1.13, CI = 1.00–1.27), presence of visible plaque (OR = 2.10, CI = 1.57–2.80), and caries activity (OR = 1.36, CI = 1.17–1.59). Second- (OR = 0.81, CI = 0.67–0.97) and third-trimester (OR = 0.79, CI = 0.65–0.96) pregnancy status were associated with lower CPITN scores compared to first-trimester pregnancy status. After adjustment, presence of visible plaque remained the main determinant of higher CPITN scores (OR = 1.88, CI = 1.37–2.59) (Table 4).

## 4. Discussion

The present study found high prevalence of gingival bleeding (84.4%) among pregnant women. Of note, our study was performed at a referral service. In a population-based study performed in the same city, the prevalence of gingivitis was lower (37.5%) among adults (24 years old) [28]. Considering pregnant and nonpregnant women, Gürsoy et al. [12] revealed that the mean percentages of bleeding on probing were constantly higher in pregnant women after five assessments. In a similar study, pregnant women were 2.2 times more likely to suffer from gingivitis compared to nonpregnant women (95% CI, 1.1–4.7) [23].

The presence of dental plaque was the strongest associated factor with gingival bleeding (OR = 2.92, CI = 1.19–4.48). Although plaque is the main factor in the gingival index [22], several studies have reported an increase in gingival inflammation during pregnancy without associated changes in plaque levels [10, 13, 15, 29–31]. The greatest prevalence of gingival bleeding was found among pregnant women in the first trimester of pregnancy. A recent meta-analysis [8] revealed lower gingival indices [5] in pregnant women in the first trimester compared to those in their second or third trimester of pregnancy in both cohort (*p* = 0.001; *p* = 0.000, resp.) and cross-sectional (*p* = 0.000; *p* = 0.030, resp.) studies. However, a comparison of these results to the present study must be considered with great caution due to methodological variability. Furthermore, considering the known effects of estrogen and progesterone on the periodontium during pregnancy [13, 17, 32–34], it is possible to assume that, at follow-up, women presenting with gingival bleeding in the first trimester would exhibit more extensive gingival tissue inflammation in the second and/or third trimesters of pregnancy.

The nonuniform distribution in pregnancy status of women seeking treatment in our referral service is one possible limitation of our study. Lower adherence was observed among women in the first trimester (*n* = 33), which may be explained by the delay in pregnancy discovery and/or barriers against attending prenatal dental visits [35, 36]. During the first trimester, some women experience nausea and vomiting in response to increased levels of human chorionic gonadotropin (hCG) hormone [37]. This fact may interfere with the frequency and proper procedure of tooth brushing, which may contribute to dental plaque accumulation. In another survey, most pregnant women reported that tooth brushing was nearly impossible, especially in premolar and molar areas, due to the pregnancy-related nausea [12]. Moreover, Taani et al. [38] reported a significant association with pregnancy-related vomiting and increased gingival inflammation in a cross-sectional study, suggesting the impaired capability for proper brushing as the main reason for this result.

Regarding the CPITN results, gingival bleeding (score 1) was reported in 29% of pregnant women in our study; similarly, the results of Toygar et al. [39] demonstrate a gingival bleeding prevalence of 24.2% in Turkish pregnant women. The presence of dental plaque was the strongest associated factor with higher levels of periodontal disease (OR = 1.88, CI = 1.37–2.59).

Low family income and low educational level were significantly associated with gingival bleeding only in the crude analysis. This result is in agreement with Vogt et al. [40], who found that 47% of low-income Brazilian pregnant women exhibited poor periodontal conditions. In another study, gingivitis was also related to professional level and level of education [3]. Low socioeconomic conditions may reflect an inaccessibility to dental clinics and unawareness of oral hygiene. In the present study, not obtaining prenatal care seemed to be a potential risk factor for gingival bleeding. Not obtaining prenatal care may suggest a lack of attention to a pregnant woman's own general health, including oral health. However, this finding should be interpreted with caution, considering the reduced number of pregnant women without prenatal care in the study sample.

Within the limits of the present study, it can be concluded that, in pregnant women, the presence of dental plaque and first-trimester pregnancy status were the main implicated factors for gingival bleeding and higher CPITN scores, after adjustments for socioeconomic and demographic factors, as well as medical and dental history.

## Figures and Tables

**Table 1:** Study population sociodemographics (*n* = 311).

Variables	Total	
	*N*	%
Skin color	288	100.0
White	216	75.0
Non-white	72	25.0

Age (years)	311	100.0
15–19	49	15.8
20–34	219	70.4
35–44	43	13.8

Education level	311	100.0
≤8 years	147	47.3
>8 years	164	52.7

Family income^*∗*^	307	100.0
≥US$1051	83	27.0
US$351–US$1050	127	41.4
≤US$350	97	31.6

^*∗*^The family income was measured in terms of the Brazilian minimum wage, which corresponds to approximately US$ 350.

**Table 2:** Clinical variables related to pregnancy (*n* = 311).

Variables	Total	
	*N*	%
Stage of pregnancy^*∗*^	311	100.0
First trimester	33	10.6
Second trimester	154	49.5
Third trimester	124	39.9

Frequency of tooth brushing	279	100.0
<1 time per day	14	5.0
1–3 times per day	200	71.7
>3 times per day	65	23.3

Frequency of flossing	311	100.0
<1 time per day	213	68.5
≥1 time per day	98	31.5

Visible plaque	266	100.0
No	49	18.4
Yes	217	81.6

Gingival bleeding	288	100.0
No	45	15.6
Yes	243	84.4

Caries activity	309	100.0
Yes	94	30.4
No	215	69.6

CPITN index	296	100.0
Score 0 (no periodontal disease)	23	7.8
Score 1 (induced gingival bleeding)	86	29.1
Score 2 (calculus)	138	46.6
Score 3 (periodontal pocket of 4 or 5 mm)	38	12.8
Score 4 (periodontal pocket ≥ 6 mm)	11	3.7

^*∗*^Stage of pregnancy at the moment that mothers arrived at Prenatal Oral Health Program.

**Table 3:** Crude and adjusted analysis [prevalence ratio (PR)] for the association between independent variables and dental gingival bleeding in pregnant women.

Variables	Gingival bleeding		Crude PR (95% CI)	*p* value	Adjusted PR (95% CI)	*p* value
	*n*	%				
Skin color						
White	165	82.5	1.00	0.004	1.00	0.885
Non-white	63	94.0	1.13 (1.04–1.24)		1.01 (1.92–1.08)	

Age (years)						
15–19	39	83.0	1.00			
20–34	172	86.0	1.03 (0.89–1.19)	0.620		
35–44	32	78.0	0.94 (0.76–1.15)	0.564		

Education level						
>8 years	126	81.3	1.00	0.116	1.00	0.371
≤8 years	117	88.0	1.08 (0.98–1.19)		1.03 (0.95–1.12)	

Family income						
≥US$1051	55	70.5	1.00		1.00	
US$351–US$1050	105	91.3	1.30 (1.11–1.52)	0.001	1.07 (0.95–1.20)	0.259
≤US$350	80	87.9	1.25 (1.06–1.47)	0.007	1.02 (0.88–1.17)	0.771

Stage of pregnancy						
First trimester	29	96.7	1.00		1.00	
Second trimester	120	84.5	0.87 (0.79–0.96)	0.007	0.89 (0.78 –1.01)	0.084
Third trimester	94	81.0	0.83 (0.75–0.93)	0.002	0.83 (0.73–0.95)	0.008

Prenatal care						
Yes	236	84.0	1.00	<0.001	1.00	0.330
No	6	100.0	1.19 (1.13–1.25)		1.13 (0.87–1.47)	

Frequency of tooth brushing						
≥3 times per day	48	80.0	1.00	0.618		
≤2 times per day	164	83.2	0.97 (0.86–1.09)			

Frequency of flossing						
≥1 time per day	166	83.8	1.00	0.705		
<1 time per day	77	85.6	1.02 (0.91–1.13)			

Visible plaque						
No	16	33.3	1.00	<0.001	1.00	<0.001
Yes	206	95.8	2.87 (1.92–4.29)		2.79 (1.81–4.30)	

Caries activity						
No	187	93.0	1.00	<0.001	1.00	0.875
Yes	56	64.4	1.44 (1.23–1.69)		1.01 (0.89–1.14)	

**Table 4:** Crude and adjusted analysis [prevalence ratio (PR)] for the association between independent variables and CPITN scores in pregnant women.

Variables	CPITN			
	Crude PR (95% CI)	*p* value	Adjusted PR (95% CI)	*p* value
Skin color				
White	1.00	**<0.001**	1.00	0.171
Non-white	1.25 (1.10–1.41)		1.10 (0.95–1.27)	

Age (years)				
15–19	1.00			
20–34	1.04 (0.86–1.25)	0.663		
35–44	1.02 (0.81–1.27)	0.855		

Education level				
>8 years	1.00	**0.035**	1.00	0.986
≤8 years	1.13 (1.00–1.27)		0.99 (0.87–1.14)	

Family income				
≥US$1051	1.00		1.00	
US$351–US$1050	1.22 (1.04–1.44)	**<0.001**	1.06 (0.89–1.25)	0.472
≤US$350	1.36 (1.16–1.60)	**0.013**	1.13 (0.94–1.36)	0.162

Stage of pregnancy				
First trimester	1.00		1.00	
Second trimester	0.81 (0.67–0.97)	**0.026**	0.86 (0.69–1.06)	0.176
Third trimester	0.79 (0.65–0.96)	**0.022**	0.80 (0.63–1.00)	0.056

Prenatal care				
Yes	1.00	0.778		
No	1.04 (0.76–1.41)			

Frequency of tooth brushing				
≥3 times per day	1.00	0.202	1.00	0.640
≤2 times per day	1.09 (0.95–1.24)		1.03 (0.88–1.21)	

Frequency of flossing				
≥1 time per day	1.00	0.810		
<1 time per day	1.01 (0.89–1.14)			

Visible plaque				
No	1.00	**<0.001**	1.00	**<0.001**
Yes	2.10 (1.57–2.80)		1.88 (1.37–2.59)	

Caries activity				
No	1.00	**<0.001**	1.00	0.594
Yes	1.36 (1.17–1.59)		1.05 (0.71–1.46)	
